# The impact of COVID-19 on the lives and mental health of Australian adolescents

**DOI:** 10.1007/s00787-021-01790-x

**Published:** 2021-04-28

**Authors:** Sophie H. Li, Joanne R. Beames, Jill M. Newby, Kate Maston, Helen Christensen, Aliza Werner-Seidler

**Affiliations:** 1 Black Dog Institute, UNSW, Hospital Road, Randwick, Sydney, 2022 Australia; 2 School of Psychology, UNSW, Sydney, NSW Australia

**Keywords:** Psychological distress, Depression, Anxiety, COVID-19, Pandemic, Adolescent health

## Abstract

There has been significant disruption to the lives and mental health of adolescents during the COVID-19 pandemic. The purpose of this study was to assess the psychological and lifestyle impact of the pandemic on Australian adolescents, using an online survey, administered during the outbreak. Self-report surveys were administered online to a sample of 760 Australian adolescents aged 12–18 years assessing impact on a range of domains including behaviour, education, relationships, lifestyle factors (exercise, technology use, and sleep), and mental health outcomes (psychological distress, loneliness, health anxiety and well-being). Results showed that three quarters of the sample experienced a worsening in mental health, since the pandemic began, with negative impacts reported on learning, friendships and family relationships. There were also high higher levels of sleep disturbance, psychological distress and health anxiety, relative to normative samples. Effects on mental health were worse among those who reported a previous diagnosis of depression and/or anxiety relative to those without no such history. Adolescents are already vulnerable to the onset of mental illness at this developmental stage, and the current research underscores the need to find rapid and accessible ways to support adolescent mental health during times of crisis.

**Supplementary Information:**

The online version contains supplementary material available at 10.1007/s00787-021-01790-x.

## Introduction

As of the 11th April 2021, there have been more than 134 million COVID-19 cases and 2.9 million deaths worldwide [1]. With 29,369 cases and 909 deaths in Australia, even considering Australia’s relatively small population size, the rate of COVID-19 infection and related deaths has remained low compared to many other countries [1]. As the pandemic and associated restrictions continue globally, there has been growing concern about the impact on mental health. Studies from around the world have shown that most individuals report increased psychological distress and worsened mental health [2–4], an effect which seems to be amplified among those with a history of mental illness [5, 6]. Although worsened mental health has been documented across the lifespan, several studies have found young adults (aged 18–24 years) are experiencing the greatest deterioration in mental health [3, 7]. Very few studies have assessed the impact of the pandemic-related disruption on adolescents [8], which is concerning, because adolescence represents a time of social transformation, marked by an increased need for peer interaction and heightened sensitivity to social stimuli [9]. Adolescents demonstrate increased independence from their families, begin to establish relationships with peers based on shared values and ideas [10], and become more sensitive to peer acceptance, approval and rejection than either children or adults [11]. Because of this sensitivity, it is likely that social distancing measures, together with school closures, may have a greater negative effect on adolescents. Support for this comes from a recent review of the literature involving more than 50,000 young people that found social isolation and loneliness significantly increases the risk of mental illness in young people [12].

Despite the significant potential impact of pandemic-related restrictions on adolescents, little empirical work has addressed this possibility [13]. Six published studies have assessed adolescent mental health (< 18 years) in response to the pandemic, three conducted in China, one each in Canada, Germany and the US [14–19], which collectively show that the prevalence of mental illness (most commonly depression and anxiety) is elevated relative to pre-pandemic prevalence estimates. Evidence of a similar pattern of worsening youth mental health in Australia is emerging. For example, the Kids Helpline (a 24-h free counselling service for 5–25 years), has reported a 40% increase in calls to the service, and the number of young people attending the hospital Emergency Department for self-harm has increased by 33%, both relative to the same period in 2019 [20, 21]. In addition, young people’s behavior and lifestyles have also been impacted by COVID-19. For example, one study [17] found that most teenagers reported engaging in at least some social distancing, and another found that 94% of adolescents reported engaging in protective behaviours, such as wearing a mask, hand washing and social distancing [15]. Moreover, worsening sleep and feelings of isolation and loneliness have been reported [22], as has disruption to learning and education [15]. Together, the available data indicates significant impact on young people’s daily lives.

Beyond these studies, it is unknown how the disruption to daily life has impacted adolescents’ behavior and worries as they relate to COVID-19. The impact on their peer relationships, family relationships, feelings of loneliness, learning and education, lifestyle factors (e.g. exercise, sleep, and technology use), and how these relate to mental health, requires investigation to understand the support adolescents require. The overall objective of the current study is to address this gap and answer calls from the scientific community to assess how young people’s lives and mental health has been impacted by the pandemic [23, 24]. The first aim of this study was to investigate the psychological impact of the pandemic on adolescents. We included measures of psychological distress, loneliness, health anxiety and wellbeing, which we expected to have worsened, as informed by the emerging literature [14, 15, 17]. Based on previous studies from the adult literature [5, 6], we expected that those who had a pre-existing history of depression and/or anxiety would show a worse psychological response to the pandemic.

The second aim was to understand how the pandemic and associated containment measures impacted the lives of adolescents. To this end, we collected data on young people’s demographic characteristics, worry about contracting COVID-19, changes to behavior, sleep disturbance, exercise, and technology use. Consistent with past studies, we expected young people to express significant concerns about contracting COVID-19, and to report changes in behavior, sleep patterns, exercise and technology use. A final exploratory aim was to examine the relationship between levels of worry about pandemic, behaviour change, uncertainty about the future, exercise, technology use, sleep, loneliness, well-being, psychological distress and health anxiety.

Data was collected from an online survey distributed via social media advertisements. While this method may not produce a representative sample of the entire population [25], it nonetheless allowed timely access to a significant number of young people, while containment measures remained in place. The recruitment period (end of June–beginning of August 2020) included the relaxing of lockdown restrictions across Australia (end of June–beginning of July), with the exception of Melbourne and the state of Victoria, which went into a second lockdown on the 8th of July (Melbourne) and 2nd of August (Victoria). As a result of these restriction differences, we also compared young people in Victoria who completed the survey during the second lockdown (on or after 8th July) to young people in the rest of the country on mental health outcomes. At the time of survey completion (31st of August 2020), there had been over 24 million COVID-19 cases and 838, 924 deaths worldwide, with 25, 547 cases and 600 deaths in Australia.

## Method

### Participants

Participants were aged 12–18 years, living in Australia. Data was collected via the Qualtrics platform from 22 June 2020 to 5 August 2020.

### Ethics approval and consent

The study was approved by the UNSW Human Research Ethics Committee (HC200334). All respondents were required to pass a Gillick Competency Task to ensure they understood the study, and had the capacity to provide informed consent, before providing consent [26].

### Measures

#### Demographics, general health and mental health history

Information was collected on participants’ age, school grade, gender, country of birth, language spoken at home, Aboriginal and Torres Strait Islander status, state of residence and who they lived with. Participants indicated if they had a parent or carer whose job had been impacted by the pandemic. Participants rated their self-rated health [27], and were asked whether they had a chronic illness, had ever been diagnosed with depression or anxiety by a professional, and current mental health treatments.

#### COVID-19 exposure, perceived risk and behaviour change

The items below were generated from a previous survey [28].

- COVID-19 exposure: participants were asked five yes/no questions about COVID-19—whether they had been tested/diagnosed, whether a family member or close contact had been diagnosed, and whether they had been required to quarantine for 14 days.
- Perceived risk: participants were asked four questions relating to their perceptions of risk and their concerns about contracting COVID-19. The first question assessed how worried they were about catching COVID-19 on a five-point scale (not at all—extremely concerned). They then rated perceived likelihood they would catch the virus on a visual analogue scale (VAS) from 0 (not at all likely) to 100 (extremely likely), and perceived behavioural control (i.e., how much they thought they could do to protect themselves from catching the virus), on a 0 (I can’t do anything) to 100 (I can do a lot) VAS. The final questions assessed perceived illness severity if they did catch COVID-19 (response options were: no symptoms, mild symptoms, moderate symptoms, severe symptoms, severe symptoms requiring hospitalisation, and severe symptoms leading to death).
- Behaviour change: Participants indicated whether they had engaged in eight social distancing and hygiene behaviours over the past week (hand washing, hand sanitising, facemask wearing, avoidance of: going to the shops/touching surfaces/spending time with people from outside the household/going to school and whether they stayed home as much as possible). Response options were on a five-point scale (1 = not at all to 5 = to all of the time).

#### COVID-19 impact on physical and mental health, school/education and relationships

- Physical and mental health: young people were asked, in two separate questions, whether their physical and mental health had been impacted by the pandemic (a lot better, a little better, stayed the same, a little worse, a lot worse).
- School and education: respondents indicated whether their school shut down during the pandemic and whether they participated in online learning (both yes/no). Those engaged in online learning reported up to three challenging aspects of completing school work at home, from a forced choice list (not enough family support, parents working and didn’t have time to help, access to technology, speed of internet, harder to learn online, too many distractions, not enough support from teachers, not motivated, other). Participants indicated how much they felt the pandemic had impacted their learning overall (positively, not at all, and negatively).
- Peer relationships: two questions assessed how the pandemic had impacted participants’ friendships; first, how socially connected they felt to their friends (more connected, no change, less connected); and second, impact on friendships overall (positively, negatively, and not at all).
- Family functioning: two items assessed young people’s family relationships, in terms of overall impact on relationships with family members (improved, no change, worsened), and impact on family stress levels (less stress, no stress, more stress).

#### Lifestyle factors

- Exercise: participants indicated the number of days they exercised for at least 30 min over the previous week (0, 1–2 days, 2–4 days, 5–6 days, every day, and don’t know), and to report whether, they exercised more, less, or the same amount as they usually would.
- Technology use: for the previous week, participants estimated total time spent in front of screens each day, not including time spent on school work (< 1 h, 1–2 h, 2–4 h, 6–8 h, and > 8 h). They also reported how much time was used to connect with friends or family (same response options) and whether their use of technology had changed since the pandemic (less than before, the same, more than before).
- Sleep: sleep was measured using the Insomnia Severity Index (ISI), which is a seven-item self-report measure of insomnia symptoms over the previous 2 weeks [29]. Responses are reported on a Likert scale from 0 to 4 with the following cut-off scores: 0–7 no clinically significant insomnia, 8–14 subthreshold insomnia, 15–21 moderate severity insomnia, and 22–28 severe insomnia [29]. The ISI has been widely administered to, and validated in, general adolescent samples [30].
- Loneliness: a single item was selected to assess loneliness and was taken from the UCLA Loneliness Scale [31]. Participants were asked to indicate how often they felt alone over the past 2 weeks (hardly ever, some of the time, and often).
- Uncertainty about the future: a single item was selected to ask participants about their feelings of uncertainty about the future, ranging from ‘not at all’ to ‘extremely’ on a five-point scale, where a higher score represents greater levels of uncertainty.

#### Mental health and well-being

The Kessler-6 (K6) assessed general psychological distress over the past 30 days [32, 33]. The K6 is a valid and reliable predictor of probable mental illness. Clinical validation studies, which define mental illness as the presence of a DSM disorder, have found the K6 has a specificity of 0.96 and a total accuracy of 0.92, and has comparable psychometric properties to clinical tools, such as the Compositive International Diagnostic Inventory in assessing the presence of probable mental illness [33]. The K6 has been validated and used in the largest population mental health survey in Australia [34] and has also been validated in adolescent samples [35], making it ideally suited to measure adolescent psychopathology in large surveys.

The seven-item Warwick Edinburgh Mental Well-Being Scale—short form (SWEMWS; [36]) assessed mental well-being over the past 2 weeks and has validated for use in young people [37]. The three-item Body Preoccupation Scale of the Illness Attitude Scales [38] was administered to assess health anxiety.

### Procedure

Participants were recruited via study information posted on the Black Dog Institute website and via paid advertisements circulated on Facebook and Instagram which targeted 12–18-year-old Australians. Participants responded to advertisements by clicking on a link which took them to the survey landing page. They read the electronic information sheet and consent form, completed the Gillick Competency Task, before accessing the survey. Upon completion, participants were placed in a draw to win one of five $50 vouchers.

### Statistical analyses

Demographic and clinical characteristics were reported using descriptive analyses. Where possible, outcomes from standardised measures were compared to normative, general population data. Independent samples *t* tests compared participants with and without a prior diagnosis of anxiety and/or depression on outcome variables (e.g. K6). Zero order correlations were conducted between worry about COVID, behaviour change, lifestyle and mental health variables.

## Results

In total, 1743 young people viewed the study page, and 945 participants provided consent and started the survey. Of those, 185 did not provide enough data (< 90% complete) to be included. The final sample comprised 760 participants.

### Demographics

Table 1 summarizes participant characteristics. Participants were 14.8 years on average, and ranged in school year from years 7–12. Most were female (72%), spoke English at home (87.7%) and were born in Australia (88.1%). Participants lived across all states and territories. Almost two-thirds of the sample (63.2%) reported living with two parents, and 50% indicated that their parent or career’s job had been impacted by the pandemic. General population data from the largest, most recent national mental health survey, the Australian Child and Adolescent Survey of Mental Health [39], indicated comparable demographic characteristics in a randomly selected sample of 6310 families. Specifically, in that survey, 87.8% were born in Australia and 68.6% lived with two parents or carers from their original family. Data on language spoken at home was not collected.

**Table 1:** Participant characteristics, clinical history and COVID-19 exposure

Participant Characteristics	*N* (%)
Age	
Age in years, mean (SD, range)	14.80 (1.26, 12–18)
12	31 (4.1)
13	101 (13.4)
14	154 (20.4)
15	214 (28.3)
16	225 (29.8)
17	22 (2.9)
18	9 (1.2)
Gender	
Male	144 (19)
Female	544 (72)
Non-binary	38 (5)
Different identity	14 (1.9)
Prefer not to say	16 (2.1)
State	
New South Wales	238 (31.5)
Victoria	266 (35.2)
Queensland	116 (15.3)
South Australia	42 (5.6)
Western Australia	53 (7)
Tasmania	20 (2.6)
Australian capital territory	18 (2.4)
Northern territory	1 (0.1)
Living situation	
Dual parent family	480 (63.5)
Single parent family	151 (20)
Blended family	98 (13)
Other (living with grandparents, sibling, other)	27 (3.6)
Place of birth	
Born in Australia	666 (88.1)
English main language spoken at home	
Yes	663 (87.7)
Aboriginal or Torres Strait Islander	
Yes	71 (9.4)
General health	
Excellent	103 (13.6)
Very good	291 (38.5)
Good	225 (29.8)
Fair	111 (14.7)
Poor	26 (3.4)
Current chronic illness status	
Yes	159 (21)
No	532 (70.4)
Unsure	54 (7.1)
Prefer not to say	11 (1.5)
Diagnosis of anxiety or depression	
Yes, depression only	26 (3.4)
Yes, anxiety only	84 (11.1)
Yes, depression and anxiety	155 (20.5)
No	401 (53)
Unsure	50 (6.6)
Prefer not to say	40 (5.3)
Current treatment for mental health problem	
Yes	210 (27.8)
No	501 (66.3)
Unsure	14 (1.9)
Prefer not to say	31 (4.1)
COVID Exposure	
Contracted COVID-19	2 (0.3)
Tested for COVID-19	112 (14.7)
Family member who contracted COVID-19	14 (1.9)
Close contact who contracted COVID-19	70 (9.2)
Required to quarantine or self-isolate for 14 days	85 (11.2)

### General health and mental health history

The mean rating for overall health was 3.44/5 (SD = 1.01), with most rating their health as either ‘good’ (29.8%) or ‘very good’ (38.5%). Twenty one percent reported a current chronic illness, 34% reported a previous diagnosis of either depression or anxiety, and 27.8% were receiving current mental health treatment (see Table 1).

### COVID exposure, perceived risk and behavior change

- Exposure: only 0.3% had a diagnosis of COVID-19. Few participants had a family member who had contracted the virus (1.9%), while just under 10% had a close contact who had the virus.
- Perceived risk: on average, young people expressed moderate levels of worry about contracting the virus (*M* = 2.27, SD = 0.97), and most (68.5%) were ‘a little concerned’ or ‘moderately concerned’ about catching the virus. Respondents thought it was relatively unlikely that they would contract COVID-19 (*M* = 25.29; SD = 19.81; scale 0–100), and reported a sense of agency that their behaviors could prevent them contracting the infection (*M* = 60.75; SD = 24.29; scale 0–100). Young people expected if they contracted the virus they would experience either no (5.3%) or mild (39.8%) symptoms, with 37.2% expecting moderate symptoms.
- Behavior change: most engaged in protective health-related behaviours over the previous week. Reports of handwashing were high, with 68.6% indicating they washed their hands thoroughly ‘all of the time’ or ‘most of the time’ as was sanitizer use (72.1% all or most of the time). Most (85.6%) respondents avoided touching objects or surfaces touched by other people to at least some extent, and 30% reported wearing a facemask. Respondents also avoided places to reduce the spread, with 82.1% avoiding the shops at least some of the time (43.3% reported avoiding shops all or most of the time), 81.3% avoiding leaving the house (48.4% always or most of the time), and 70.4% avoiding spending time with people outside their household (35.1% always or most of the time). Half reported not going to school in the previous week due to the pandemic.

### COVID-19 impact

**Fig. 1:**
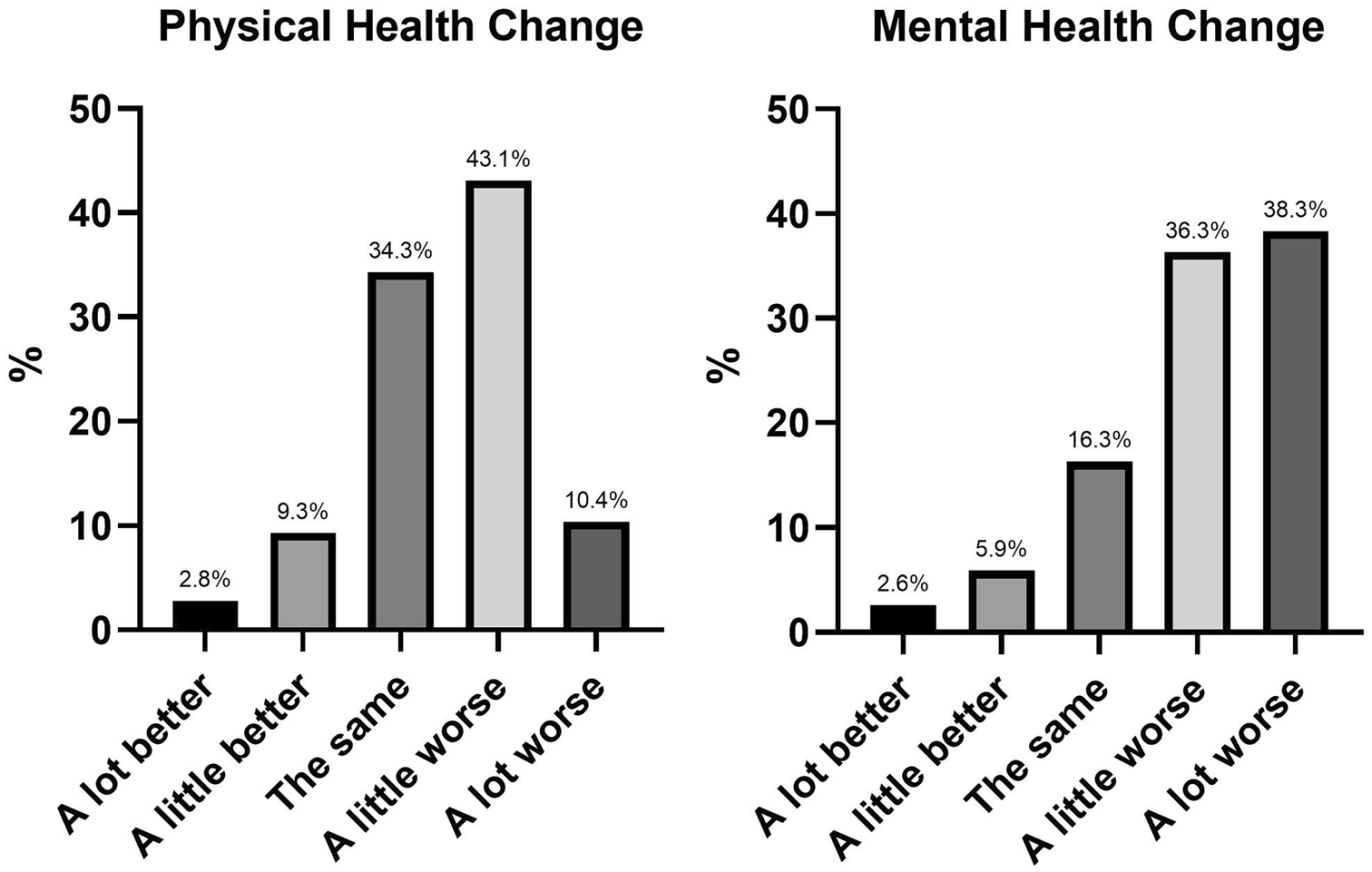
Physical and mental health change since the pandemic began

**Fig. 2:**
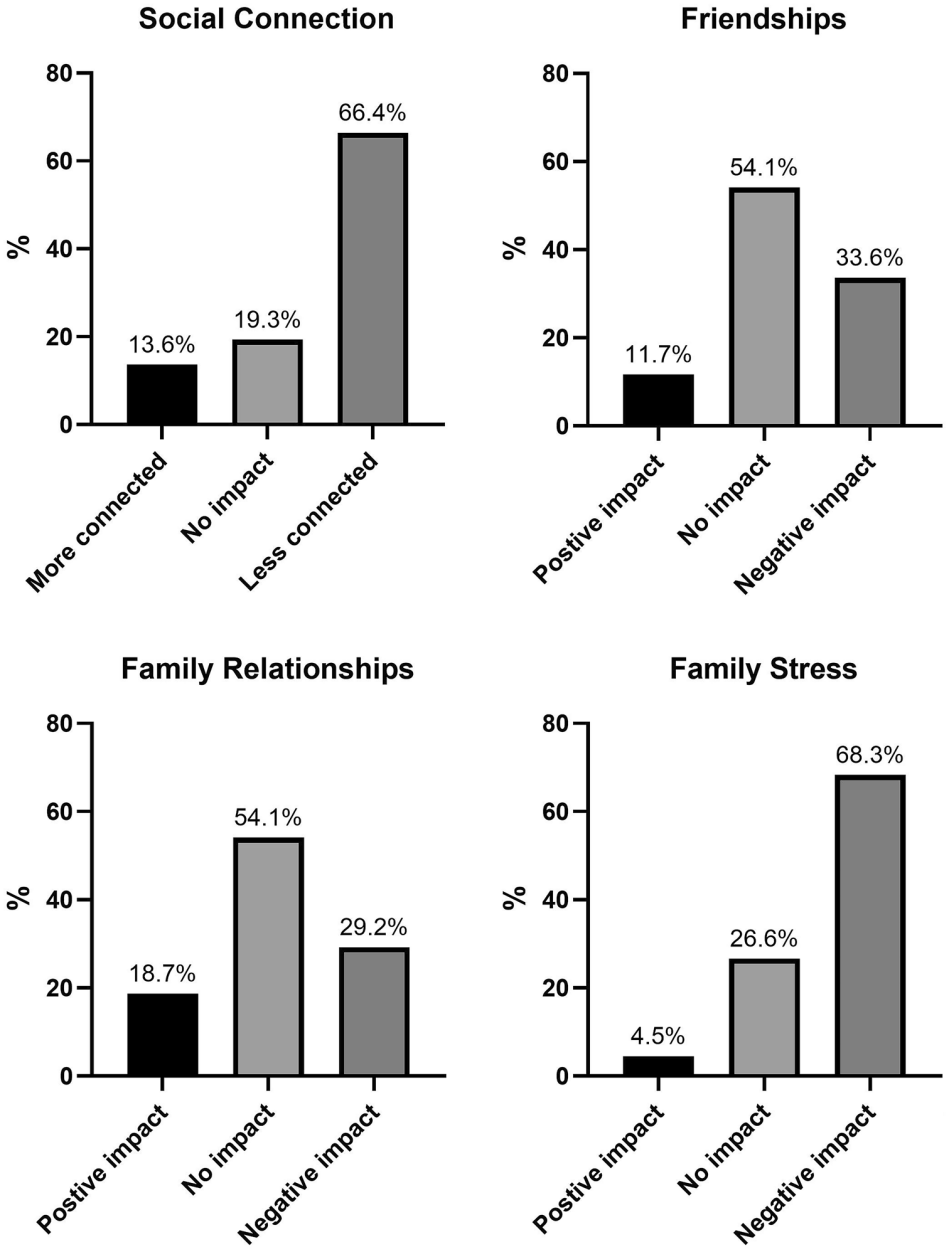
Impact of pandemic on peer and family relationships

- Physical and mental health: see Fig. 1. More than half of the participants indicated that their physical health had worsened during the pandemic. Approximately a third reported no change, and few reported an improvement. The impact of the pandemic on mental health revealed different results; most (75%) young people reported a negative effect on their mental health. Few reported no change or an improvement.
- School and education: most (87%) reported that their schools closed during the pandemic, and the majority (95.1%) engaged in online learning instead. The top three challenges to online learning were: (i) a lack of motivation; (ii) too many distractions at home; and (iii) it being more difficult to learn via online platform relative to face-to-face. A lack of support from schoolteachers, increased school workload, feelings of loneliness and slow internet was also noted. Overall, two in three young people (62.6%) felt that the pandemic had negatively impacted their learning, with 22% indicating no change and 14.9% reporting a positive impact.
- Peer relationships: see Fig. 2. Most respondents reported feeling less connected to their friends. There seemed to be a degree of stability to adolescents’ friendships, with about half of the sample reporting no overall impact on their friendships.
- Family functioning: see Fig. 2. Approximately one third of respondents reported a worsening of family relationships, and most young people reported a worsening of family stress.

### Lifestyle factors

See Table 2.

- Exercise: twenty percent reported zero instances of at least 30-min exercise during the previous week. Approximately half of respondents indicated that they exercised for 30 min on at least 1–2 days. Most young people reported either a decrease in exercise, since the pandemic began, or no change.
- Technology use: most young people reported either 2–4 h or 4–6 h daily screen use. There was a still a significant proportion of young people reporting higher levels of use (> 8 h) each day, with 72% reporting increased technology use to connect with others, usually spending around 4-h online for interaction. Almost three quarters of the sample reported increased technology use since the start of the pandemic.
- Sleep disturbance: the mean score on the ISI was 12.09 (SD = 6.03). Just under half of the sample (40.5%) reported subthreshold insomnia, while more than a quarter (28.7%) reported moderate severity insomnia and a further 6.4% reporting severe insomnia.
- Loneliness: Just over half (51.4%) of the sample reported frequently experiencing feelings of loneliness. A further third reported feeling alone some of the time.
- Uncertainty about the future: ninety-three percent of respondents reported some degree of uncertainty about the future, with approximately one third (33.8%) reporting very or extreme levels of uncertainty.

**Table 2:** Frequency and descriptive data for lifestyle and mental health outcomes

Lifestyle and Mental Health Outcomes	*N* (%)
Exercise (# ≥ 30 min exercise sessions in past week)	
None	154 (20.3)
1–2 days	236 (31.1)
3–4 days	169 (22.2)
5–6 days	102 (13.4)
7 + days	76 (10)
Exercise change due to the pandemic	
Less than usual	308 (40.5)
Same as usual	326 (42.9)
More than usual	121 (15.9)
Technology use (time spent on screens each day for non-school purposes)	
< 1 h	11 (1.4)
1–2 h	33 (4.3)
2–4 h	178 (23.4)
4–6 h	241 (31.7)
6–8 h	151 (19.9)
> 8 h	141 (18.6)
Technology use—social connection (amount of time spent connecting with friends or family)	
< 1 h	173 (22.8)
1–2 h	243 (32)
2–4 h	196 (25.8)
4–6 h	96 (12.6)
6–8 h	23 (3)
> 8 h	24 (3.2)
Technology use change due to the pandemic	
Less than usual	81 (10.7)
Same as usual	128 (16.8)
More than usual	547 (72)
Loneliness (frequency of feeling alone)	
Hardly ever	130 (17.1)
Some of the time	233 (30.7)
Often	391(51.4)
Uncertainty about the future	
Not at all	54 (7.1)
A little uncertain	218 (28.7)
Moderately uncertain	227 (29.9)
Very uncertain	158 (20.8)
Extremely uncertain	99 (13)

Standardised questionnaires	*M* (SD)	Range
Insomnia Severity Index	12.09 (6.03)	0–28
Short Warwick–Edinburgh Mental Well-being Scale	18.97 (5.87)	7–35
Kessler-6 Scale (psychological distress)	18.08 (6.63)	6–30
Illness Preoccupation Scale (health anxiety)	4.72 (4.72)	0–12

### Mental health and well-being

- Mental health: The mean score on the K6 was 18.08 (SD = 6.63; range 6–30), with just under half of the sample (48.3%) scoring above the threshold that indicates psychological distress indicative of mental illness.
- Well-being: Mean well-being score was 18.79 (SD = 5.87; range 7–35), with a higher score indicating greater levels of well-being.
- Health anxiety: on the screening survey for health anxiety, the Body Preoccupation Scale of the Illness Attitude Scales, 40.1% of young people scored above the clinical cutoff which indicates severe health anxiety.

### Comparison of mental health outcomes between young people in Victoria during the second lockdown and young people from the rest of Australia

Given that a second lockdown in Victoria was announced on the 8th of July, mid-way during the survey data collection period, we examined the mental health outcomes among young people located in this state who responded to the survey on or after this date (*n* = 116), relative to respondents across the rest of Australia, or in Victoria prior to this date (*n* = 514). We found no significant differences in mental health outcomes including loneliness, [*t *(628) = − 1.85, *p* > 0.05], psychological distress (*t* < 1), well-being (*t* < 1), or health anxiety, (*t* < 1). Accordingly, data was collapsed and not segregated by region for the analysis.

### Comparison between adolescents with and without a prior mental health diagnosis

Young people with and without a self-reported diagnosis of depression and/or anxiety were compared on outcomes (see Table 3). After removing those who did not know if they had a diagnosis (6% of the overall participant pool) or chose not to say (5%), 60% percent of the remaining sample reported no history of mental illness, and 40% reported a previous diagnosis of depression or anxiety (3.9% depression only, 12.6% anxiety only, and 23.3% both depression and anxiety). These categories were collapsed for the subsequent analyses into presence or absence of a diagnosed depression and/or anxiety. Participants with a history of depression or anxiety reported worse mental and physical health on all measures (see Table 3), and reported lower levels of exercise, great use of technology, poorer sleep, higher levels of loneliness, uncertainty about the future, psychological distress, health anxiety and lower levels of well-being (all *p*s < 0.05; see Table 3 for full statistics).

**Table 3:** Comparison between respondents with and without a self-reported mental health diagnosis of depression and/or anxiety

	Prior mental health diagnosis			No prior mental health diagnosis			Independent samples *t* test
	*N*	Mean	SD	*N*	Mean	SD	
Perceived risk	264	23.89	19.02	399	23.89	19.02	*t* (661) = − 1.74, *p* = 0.08
Perceived control	264	60.03	25.47	399	61.34	24.05	*t* (661) = 0.67, *p* = 0.51
Worry about catching COVID-19	265	2.39	1.04	401	2.20	0.89	*t* (664) = − 2.53, *p* < 0.01^*^
Severity of illness	261	1.91	1.09	397	1.59	0.87	*t* (656) = − 4.17, *p* < 0.01^*^
Change in physical health	265	2.44	0.91	401	2.61	0.91	*t* (664) = 2.31, *p* < 0.05^*^
Change in mental health	265	1.93	1.09	401	2.08	0.91	*t* (664) = 1.80, *p* = 0.07
Exercise	259	1.40	1.23	393	1.74	1.24	*t* (649) = 3.40, *p* < 0.01^*^
Technology use	264	3.37	1.21	401	3.06	1.16	*t* (663) = − 3.28, *p* < 0.01^*^
Sleep	264	14.49	5.70	401	10.15	5.62	*t* (662) = − 9.72, *p* < 0.01^*^
Loneliness	264	5.63	2.30	401	4.56	2.44	*t* (663) = − 5.61, *p* < 0.01^*^
Uncertainty about the future	265	3.38	1.13	401	2.78	1.08	*t* (664) = − 6.88, *p* < 0.00^*^
Psychological distress	265	20.23	6.24	401	16.33	6.64	*t* (664) = − 7.69, *p* < 0.01^*^
Health anxiety	265	5.50	2.88	400	4.10	2.66	*t* (662) = − 6.40, *p* < 0.01^*^
Well-being	265	16.98	5.10	401	20.77	5.87	*t* (664) = 8.58, *p* < 0.01^*^

### Association between COVID-fears, behaviour, lifestyle factors and mental health

See Supplementary materials for results. There was a significant positive correlation between fears about contracting COVID-19, and behaviour change, feelings of uncertainty about the future, poor sleep, psychological distress, and health anxiety (all *p*s < 0.01). Greater exercise was associated with lower levels of screen time, better sleep, lower levels of psychological distress and a greater sense of well-being (*p*s < 0.01). Overall screen time was associated with more psychological distress; however, when screen time was used to connect with friends and family, the relationship with distress was no longer evident, and increased screen time for connection was associated with lower levels of loneliness and higher levels of well-being (*p*s < 0.01). Finally, as expected, all the mental health variables were significantly associated, with positive correlations between loneliness, health anxiety and psychological distress, and an inverse association between these variables and well-being being detected.

## Discussion

This study is novel in its assessment of a broad range of variables that relate not only to psychopathology, but also to young people’s experiences of COVID-19, the impact on their learning and relationships, as well as a range of lifestyle factors, including sleep, exercise and screen time. To our knowledge, existing adolescent studies focus on the mental health impact of the pandemic on young people (e.g. [12, 18, 19]) or lifestyle factors (e.g. [40]) or worry, concern and behaviour change related to COVID-19 (e.g. [17]), but not these factors collectively. Assessing these variables in the same sample is necessary to form a rich and complete picture of the disruption to young people’s lives, mental health and well-being. The benefit of this approach is demonstrated by our findings; for example, we demonstrate, for the first time, that the use of technology for the purposes of social connection (rather than just technology use per se) is associated with a greater sense of well-being and lower levels of loneliness. This important advance in knowledge can help guide the way teenagers can be encouraged to use technology to maintain social connection, when face-to-face interactions are not possible.

The results of our study also showed that adolescents with a self-reported history of depression or anxiety experienced heightened levels of loneliness, greater trouble sleeping, more uncertainty about the future, higher levels of health anxiety, greater psychological distress and lower levels of well-being, relative to those without a depression or anxiety history, in response to the pandemic. The proportion of participants reporting a history of depression or anxiety was comparable to lifetime prevalence estimates for adolescents [41], suggesting respondents were broadly representative of the general population in terms of psychiatric history. This is the first finding to indicate that Australian adolescents with a history of depression or anxiety experience greater levels of psychopathology and disruption to daily life in response to a crisis; in this case, the COVID-19 pandemic. Moreover, these results are consistent with findings examining adolescents in Canada [18] and adults in Australia and the US [5, 6], which collectively suggest that a history of mental illness is a vulnerability factor for deterioration in mental health during the pandemic.

There are important implications that follow on from this finding. First, from a practical perspective, the knowledge that challenges, such as a pandemic, lead to an exacerbation of mental illness among those with a history of anxiety and/or depression suggests that psychological treatments for these disorders could incorporate symptom management plans for challenging situations likely to exacerbate symptoms, as a component of therapy. Moreover, an immediate and much needed policy change is needed to expand existing mental health services for young people. There has already been an increased burden on these services [21] and for services to continue to provide care to young people with exacerbations in existing mental health problems, while simultaneously supporting those presenting to services for the first time, an expansion of such services is imperative. Evaluations of the impact of the pandemic on existing services and their response to the crisis (e.g. [42, 43]) may guide such an expansion and inform policy to deal with future periods of increased mental health service need. Similarly, the placement of clinically trained staff into schools to support students either face-to-face when possible, or via telehealth when not, would do much to reduce the burden on external services. Finally, another method to reduce overburdening existing services is to consider the use of effective digital mental health interventions, and blended modalities that combine digital with clinician support, which will likely improve the efficiency with which young people with mental health problems can be supported [44]. In the longer term, a greater focus on the prevention of mental illness and upskilling young people with effective strategies to manage their mental health, especially at times of vulnerability, will have a downstream impact on ensuring services do not become overburdened, particularly during times of crisis. Taken together, the findings reported in this study underscore the need for a proactive mental health response to support young people through this tumultuous and disruptive time in their lives, through changes at both the practice and policy level.

Although respondents had little direct or indirect experience with COVID-19, more than three quarters were worried about contracting the virus, replicating findings with adults [6] and adolescents [15]. Most young people believed they could reduce their risk of contracting COVID-19 and engaged in behaviors to lower their risk (e.g. handwashing and social distancing), in contrast to common portrayals by the media suggesting young people are not compliant with restrictions (e.g. [45]). Our results showed that worry about contracting COVID-19 was associated with greater levels of overall behaviour change, demonstrating a practical benefit of some degree of worry.

This study also sheds light on the disruption and impact of the pandemic on young people’s lives. Almost the whole sample (> 95%) had engaged in online learning, and most reported a negative impact. At the time our survey was undertaken, schools and families had to adapt to online learning from home with little-to-no preparation time. While meta-analyses have shown that with optimal delivery and support, online formats can be as effective as face-to-face in terms of learning outcomes for adults [46], there is no evidence to suggest this is the case for young people. Indeed, because school education is primarily face-to-face, data about the effectiveness of online schooling is lacking. Online learning requires a greater level of independence, motivation, and discipline than classroom learning, and these are skills which young people may not have fully developed [47].

Participants reported a negative effect of the pandemic on friendships, and feelings of loneliness, which were associated with higher psychological distress and lower well-being. Adolescents are at a crucial stage of development involving the formation of a sense of self and identity through shared interests and values with their peers [48]. Given a lack of social connection has negative consequences on social and cognitive development [9], and loneliness increases the risk of the development of depression and other disorders [12], mental health prevention and intervention efforts need to focus on improving social connection, particularly in areas that have containment measures in place for prolonged periods. Until restrictions are lifted, the use of technology to connect with others might mitigate the potential disruption to adolescents’ social needs. With widespread smartphone use [49], it is reassuring that studies have found that core components of quality face-to-face interactions can be replicated online [50]. Consistent with expectations, our study found that nearly three quarters of participants reported increased use of technology to connect with others and this was associated with lower levels of loneliness and a greater sense of well-being. This finding aligns with research from China showing increased smartphone and social media use at the height of the pandemic [16]. Most adolescents in our study reported spending about 4 h a day connecting with others online. Whether this will mitigate potential long-term consequences of social deprivation associated with lockdowns will need to be addressed by future longitudinal studies.

Most respondents indicated that the pandemic had increased stress levels within their family and half reported an impact on the job of a parent or carer. Viewed in this context, together with adjusting to online learning and the requirement for parents to manage their own professional responsibilities with caring and supporting their child’s learning, it is not surprising this has been a stressful time for families [51]. This aligns with findings from around the world that are beginning to highlight higher levels of stress and mental ill-health experienced by certain sub-groups of the population, including women, who are more likely to be in caring roles, as well as parents and young people [3, 7]. Despite this increased family stress, > 50% of adolescents indicated that their relationships with family members had remained unchanged, and an additional 18.7% reported an improvement; something that has not previously been found in an adolescent sample. It could be the case that in some families, more time at home with family members has some advantages, and feeling more connected to loved ones is a benefit of COVID-19 that has been found in adults [52].

Encouragingly, > 50% of the adolescents continued with or increased their regular levels of exercise. There are well-documented links between exercise and reduced risk of depression and anxiety across the lifespan [53] and the relationship between exercise and lower levels of psychological distress and great well-being in our sample is consistent with this broader literature [16]. Given that even the strictest lockdowns in Australia have allowed for up to 1 h of exercise per day, exercise could be promoted in public health campaigns to prevent deteriorating mental health should the lockdown and period of restrictions continue. Relatedly, a significant proportion of adolescents also reported increased difficulty sleeping, and exercise is one way in which sleep quality and duration can be improved [54], which was supported by our correlational analyses.

Of concern was that about half of the sample reported a worsening in their physical health, since the pandemic began, while 75% reported a negative effect on their mental health. The worsening of mental health in our sample was markedly consistent to that of a recent adult Australian survey [6], which found that 78% of their respondents had reported worsened mental health. Overall, adolescents reported greater psychological distress and lower levels of well-being relative to normative data available from population surveys conducted prior to the pandemic [55, 56], with rates of psychological distress indicative of probable mental illness increasing almost twofold from 24.3% before the pandemic [56], to 48.3% in this survey. This finding accords with studies from China and Germany showing elevated mental illness in young people [8, 19]. It is important to note that the K6 is not a diagnostic interview and so without information about the exact duration of the symptoms, degree of interference in daily life and distress to the individual, drawing diagnostic conclusions is not possible. We also cannot rule out the heightened levels of psychological distress as being at least in part attributable to sampling bias, inherent with online surveys. With these limitations in mind, our data nonetheless suggest that there are significantly elevated rates of psychological distress [56], providing insight into the acute effects of the COVID-19 pandemic on adolescent mental health.

There are several study limitations. First, as a convenience sample recruited online due to the ease of rapid administration, 72% were female, limiting the generalizability of findings to the broader adolescent population. The importance of sampling approach has been noted as a key concern during COVID-19 [25]. Follow-up studies should use diagnostic assessments, to provide an independent assessment of mental health. The study was cross sectional and so causal conclusions cannot be drawn.

Despite these limitations, this study has provided insight into the impact of the COVID-19 pandemic on multiple facets of young people’s lives. Marked deterioration in the mental health of adolescents, evidenced by three quarters of respondents reporting a negative impact of the pandemic on mental health and almost half reporting psychological distress levels indicative of a probable mental illness (a twofold increase from pre-pandemic levels), emphasises the need for adequate infrastructure to support the mental health and recovery of this already vulnerable population. This study showed worse outcomes for those with pre-existing mental health conditions, further indicating the need for adequate services during times of crisis, but also the need for current treatments to provide young people with skills to manage their mental health during times of adversity.

## Supplementary Information

Below is the link to the electronic supplementary material.

Supplementary file1 (DOCX 18 KB)

## Data Availability

Not publicly available due to the sensitive nature of the data and ethical guidelines.
