# Supplementary material for: The impact of COVID-19 on the lives and mental health of Australian adolescents
Source: Eur Child Adolesc Psychiatry. 2021 Apr 28;31(9):1465–77. doi: 10.1007/s00787-021-01790-x (PMC8080862; doi:10.1007/s00787-021-01790-x)
Supplement: Supplementary file 1 — Supplementary file1 (DOCX 18 KB) [file 787_2021_1790_MOESM1_ESM.docx]

Supplementary Table: Correlational Table including COVID variables, lifestyle factors and mental health and wellbeing

|  | **COVID Worry** | **Behavior Change** | **Uncertainty** | **Exercise** | **Screen Time** | **Screen Connection** | **Sleep** | **Loneliness** | **Wellbeing** | **Health Anxiety** | **Distress** |
| --- | --- | --- | --- | --- | --- | --- | --- | --- | --- | --- | --- |
| ***COVID Variables*** |  |  |  |  |  |  |  |  |  |  |  |
| Worry about contracting COVID | - |  |  |  |  |  |  |  |  |  |  |
| Behavior change | .33** | - |  |  |  |  |  |  |  |  |  |
|  |  |  |  |  |  |  |  |  |  |  |  |
| ***Lifestyle and other factors*** |  |  |  |  |  |  |  |  |  |  |  |
| Uncertainty about the future | .32** | .13** | - |  |  |  |  |  |  |  |  |
| Exercise | -.02 | -.01 | -.13** | - |  |  |  |  |  |  |  |
| Overall screen time | .01 | .13** | .18** | -.20** | - |  |  |  |  |  |  |
| Screen time to connect with others | .02 | -.01 | -.01 | .06 | .05 | -.05 |  |  |  |  |  |
| Sleep | .11** | .15** | .32** | -.10** | .17** | -.05 | - |  |  |  |  |
|  |  |  |  |  |  |  |  |  |  |  |  |
| ***Mental Health & Wellbeing*** |  |  |  |  |  |  |  |  |  |  |  |
| Loneliness | .07 | .17** | .28** | -.13** | .11** | -.19** | .31** | - |  |  |  |
| Wellbeing | -.08* | -.07 | -.41** | .21** | -.18** | .14** | -.52** | -.59** | - |  |  |
| Health Anxiety | .23** | .09 | .26** | -.06 | .02 | -.01 | .20** | .20** | -.17** | - |  |
| Psychological Distress | .17** | .15** | .44** | -.12** | .44** | -.04 | .53** | .53** | -.63** | .28* | - |

*Note:* Zero order correlations presented. ** = significant at .01 level; * = significant at .05 level.
